# Organelle Genetics in Plants 2.0

**DOI:** 10.3390/ijms241512128

**Published:** 2023-07-28

**Authors:** Pedro Robles, Víctor Quesada

**Affiliations:** Instituto de Bioingeniería, Campus de Elche, Universidad Miguel Hernández, 03202 Elche, Spain; probles@umh.es

Most of the DNA of eukaryotes is located in the nucleus. However, the chloroplasts in photosynthetic organisms, and the mitochondria in the vast majority of eukaryotes also contain part of the DNA of a eukaryotic cell. This genetic material constitutes the genomes of chloroplasts and mitochondria, also known as plastomes and mitogenomes, respectively, whose organization and inheritance patterns substantially differ from those of nuclear DNA. Extensive phylogenetic analyses clearly support the hypothesis that chloroplasts and mitochondria derive from ancestral cyanobacteria and α-proteobacteria, respectively, which were engulfed by a primitive nucleated cell and ended up establishing an endosymbiotic relationship with the host cell [1,2,3].

The correct functioning of chloroplasts and mitochondria requires hundreds of proteins. However, plastomes and mitogenomes harbor only a few dozen coding-protein genes, which are transcribed and translated in the organelle. Therefore, the overwhelming majority of the proteins that act on chloroplasts and mitochondria are encoded by the nuclear genome, and must be synthesized in the cytoplasm and subsequently transported to their target organelle. This is a consequence of the transfer throughout evolution of the genes of ancestral prokaryotes to the nucleus of the primitive host cell [1,2,3]. As a result, the expression of current nuclear and organelle genomes must be very tightly coordinated [4,5].

This Special Issue is the continuation of the previous Special Issue “Organelle Genetics in Plants”, of which we were also guest editors. It contains four original research articles, one review, and one perspective article, published by field experts. The original research articles analyze mitogenome evolution in Rubiaceae along with *Damnacanthus indicus* [6], the genomic structure of the plastome and mitogenome of *Valeriana sambucifolia* f. *dageletiana* [7], a novel P-type pentatricopeptide repeat (PPR) factor involved in chloroplast development in *Arabidopsis thaliana* cotyledons [8] and plastid transmission in *Passiflora* [9]. The review article focus on the role of massive mitochondrial fusion (MMF or hyperfusion) in plants [10], whereas the perspective work investigates the impact of the amino acid changes due to RNA editing on the structure and function of several plant mitochondria respiratory complexes [11]. In this editorial, we summarize the most relevant findings of these insightful works.

In the research article by Han et al. [6], the mitogenomes of six *Damnacanthus indicus* (Rubiaceae, Rubioideae) individuals, representing two varieties (var. *indicus* and var. *microphyllus*), were assembled. The authors performed a thorough analysis of the main features of these mitogenomes. Along these lines, the gene and intron contents of *D. indicus* were compared to the mitogenomes from representative angiosperm species. Nine genes were missing (*rpl2*, *rpl10*, *rpl16*, *rps2*, *rps7*, *rps11*, *rps14*, *rps19,* and *sdh3*) in *D. indicus*, and the gene loss pattern of *rps7* was reconstructed through a phylogenetic tree for Rubiaceae. The mitogenomes of *D. indicus* contained 36 genes of plastid origin and an intron analysis revealed a shift from *cis* to *trans* splicing of a *nad1* intron (nad1i728) in *D. indicus*, which was shared with other four Rubioideae taxa. Moreover, the mitogenome structural rearrangement in *D. indicus* was analyzed and two distinct structures, type A and B, were identified. Han et al. [6] proposed a two-step direct repeat-mediated recombination to explain the structural changes between type A and B mitogenomes. Finally, intraspecific mitochondrial DNA divergence in *D. indicus* was also analyzed by separating the six studied individuals into two subgroups, which diverged by 158 mutational steps.

In [7], Kim and Kim conducted a comprehensive study of the plastome and mitogenome sequences of the therapeutic plant *Valeriana sambucifolia* f. *dageletiana* endemic to Korea, and found a dynamic gene transfer among the three plant genomes. The plastome spanned 155,179 bp, which was slightly smaller than its counterpart in *Valeriana officinalis*. Notably, it contained eight non-plastome regions (NPRs), six of which did not match any nucleotide sequence. Database searches unveiled that one NPR exhibited some similarities with the sequences present in animal genomes, predominantly those of bony fishes. The authors hypothesized that these sequences could indicate the occurrence of gene transfer from a bony fish to the chloroplast genome of the ancestor of *V. sambucifolia* f. *dageletiana*, likely mediated by fungi or bacteria. Regarding the mitochondrial genome, this study marked the first report for a plant within the order Dipsacales. With a length of 1,187,459 bp, a substantial portion (over 30% of the entire mitogenome) did not correspond to the sequences found in the mitogenomes of terrestrial plants, perhaps as a consequence of the gene transfer from the nucleus or the chloroplast to mitochondria. While the first hypothesis was challenging to prove due to a lack of nuclear genome sequences reported in Valeriana species, the authors demonstrated its likelihood by showing that this usually happens in other plant species with similar-sized mitogenomes to that of Valeriana. Regarding the gene transfer between both organelles, Kim and Kim found 162 regions in the mitogenome of *V. sambucifolia* f. *dageletiana,* which corresponded to terrestrial plant plastomes, most of which were considered to be translocated from the plastome to the mitogenome.

The research article by Wang et al. [8] studied the function of a novel Arabidopsis P-type pentatricopeptide repeat (PPR) protein, dubbed ALBINO COTYLEDON MUTANT1 (ACM1), by screening a series of T-DNA insertion lines putatively affected in genes related to chloroplast development. The *acm1* knock-out mutant exhibited an albino seedling lethal phenotype, which suggests that *acm1* was a null allele, and fluorescent protein analysis revealed that ACM1 was a chloroplast-localized protein. To further investigate ACM1 functions by avoiding *acm1* lethality, the authors [8] created a series of RNAi lines to obtain *ACM1* knock-down transgenic plants and chose one of them for further studies. The plants from the selected RNAi line showed white cotyledons, green true leaves, and normal growth. The thorough characterization of this RNAi line revealed that *ACM1* knock-down significantly affected (i) chloroplast development in cotyledons, (ii) the accumulation of chlorophylls and photosynthetic proteins, (iii) the splicing efficiency of several group II introns in cotyledon chloroplasts, (iv) the transcript levels of plastid genes, especially those transcribed by Plastid Encoded Polymerase (PEP), and (v) the accumulation of chloroplast rRNAs and ribosome subunit protein RPS14. Taken together, the results showed that the ACM1 PPR factor played a fundamental role in early chloroplast development in Arabidopsis cotyledons.

Shrestha et al. shed light in their research article [9] on plastid inheritance in the genus *Passiflora*. While most angiosperms exhibit maternal plastid inheritance, *Passiflora* displays unique parental or biparental inheritance patterns, but this variation was uncertain due to limited previous analyses. To clarify this, the authors performed 45 interspecific crosses by involving plants from three *Passiflora* subgenera: *Passiflora*, *Decaloba* and *Astrophea*. Employing PCR and restriction analyses of amplicons, they not only determined the plastid inheritance pattern in hybrids, but also observed changes in plastid type retention throughout the plant’s life cycle. The *Passiflora* and *Astrophea* subgenera predominantly inherited paternal plastids, with occasional instances of biparental inheritance in the former. In contrast, *Decaloba* showed predominantly maternal and biparental plastid inheritance. Even in cases in which biparental inheritance was observed in hybrids, heteroplasmy was present only in cotyledons and first leaves, while mature plants seemed to retain a single parental plastid type.

Mitochondria are pleomorphic and dynamic organelles whose morphologies are important for understanding several mitochondrial cell biology aspects. In a plant mitochondria population, it is feasible to find some containing an incomplete genome or even no DNA. This DNA deficiency can be solved by fusion and the later fission of mitochondria, and fusion may involve only pairs or many mitochondria, and can even be massive (hyperfusion) [12]. Therefore, the fusion and fission of plant mitochondria are fundamental for mitogenome integrity and quality. In his comprehensive review, Rose [10] summarized our current understanding of the cell and molecular biology of mitochondria fission and fusion in plants. The author focused first on the proteins of the mitochondrial fission and fusion machineries. Along these lines, the mechanistic insight of plant mitochondrial fission is better understood than fusion. Accordingly, the role of key proteins in mitochondrial fission, such as DRP3A and 3B, is fairly well-known. Rose [10] then discussed the significance of the mitochondrial fusion/fission cycle and analyzed MMF fusion in the plant life cycle. Interestingly, he reported that MMF usually took place at critical plant life cycle moments (e.g., at developmental transitions) might be to meet their high energy request. He emphasized the importance of plant MMF in genome repair, the conservation of critical genes and plant regeneration. The author remarked that understanding the contribution of the endoplasmic reticulum and the cytoskeleton in mitochondrial fusion and fission requires further research.

RNA editing changes the sequences of coding and non-coding regions of RNA molecules and this modification can significantly impact gene expression. In land plants, wide RNA editing occurs in chloroplasts and mitochondria and perturbation of RNA editing, e.g., due to mutations in PPR proteins involved in this process, can lead to severe phenotypes, such as pollen abortion, seed development defects, and growth retardation in plants [13]. In the perspective article by Maldonado et al. [11], the authors structurally characterized the RNA-editing sites of the mitochondrially encoded subunits of plant respiratory-chain complexes for which high-resolution cryoEM structures are available: complex I, complex III2, and complex IV. The main purposes of this work were to analyze the consequences of the amino acid changes due to RNA editing in terms of their location and biochemical properties, and to show that a structural perspective can be useful for bridging the gap between sequence and phenotype. In [11] 275 edited sites were identified in the three respiratory chain-complexes analyzed across 17 plant species, and found that nearly all of them had an impact on the structure and, consequently, the function, of these protein complexes. The authors also examined the existing literature and selected some plant mutants defective in RNA-editing due to mutations in PPR proteins to investigate the structural and functional consequences of mutations in single- or multi-site editing PPR proteins. Maldonado et al. [11] demonstrate that the structural analysis of RNA-editing sites can explain the phenotypes of these RNA-editing mutants, which provides a framework for further analyses of other mutants affected in mitochondria or chloroplast RNA editing.

Taken together, the papers published in this Special Issue represent a significant advance in the current knowledge of plant organelle genetics. We wish to thank the authors of these works for their contributions and the reviewers for their critical comments, which have helped to further improve their quality. Finally, we would also like to thank Mr. Jerry Wang for providing us with the opportunity to be the guest editors of the Special Issue “Organelle Genetics in Plants 2.0”.
